# Epidemiology and One-Year Follow-Up of Neonates with CDH-Data from Health Insurance Claims in Germany

**DOI:** 10.3390/children8020160

**Published:** 2021-02-20

**Authors:** Boris Wittekindt, Nora Doberschuetz, Andrea Schmedding, Till-Martin Theilen, Rolf Schloesser, Stefan Gfroerer, Udo Rolle

**Affiliations:** 1 Department of Neonatology, University Hospital, Goethe-University, 60590 Frankfurt, Germany; nora.doberschuetz@kgu.de (N.D.); rolf.schloesser@kgu.de (R.S.); 2 Department of Paediatric Surgery and Paediatric Urology, University Hospital, Goethe-University, 60590 Frankfurt, Germany; andrea.schmedding@kgu.de (A.S.); till-martin.theilen@kgu.de (T.-M.T.); udo.rolle@kgu.de (U.R.); 3 Department of Paediatric Surgery, Helios-Klinikum, 13125 Berlin, Germany; Stefan.Gfroerer@helios-gesundheit.de

**Keywords:** congenital diaphragmatic hernia, mortality, ECMO, insurance data, follow-up

## Abstract

Congenital diaphragmatic hernia (CDH) is a major congenital malformation with high mortality. Outcome data on larger unselected patient groups in Germany are unavailable as there is no registry for CDH. Therefore, routine data from the largest German health insurance fund were analyzed for the years 2009–2013. Main outcome measures were incidence, survival and length of hospital stay. Follow-up was 12 months. 285 patients were included. The incidence of CDH was 2.73 per 10,000 live births. Overall mortality was 30.2%. A total of 72.1% of the fatalities occurred before surgery. Highest mortality (64%) was noted in patients who were admitted to specialized care later as the first day of life. Patients receiving surgical repair had a better prognosis (mortality: 10.8%). A total of 67 patients (23.5%) were treated with ECMO with a mortality of 41.8%. The median cumulative hospital stay among one-year survivors was 40 days and differed between ECMO- and non-ECMO-treated patients (91 vs. 32.5 days, *p* < 0.001). This is the largest German cohort study of CDH patients with a one-year follow-up. The ECMO subgroup showed a higher mortality. Another important finding is that delayed treatment in specialized care increases mortality. Prospective clinical registries are needed to elucidate the treatment outcomes in detail.

## 1. Introduction

Congenital diaphragmatic hernia is one of the major live-threatening congenital malformations, which requires the full spectrum of perinatal care, including prenatal diagnosis, probable prenatal therapy, postnatal intensive care and paediatric surgery. CDH has an incidence of 2.3–2.81 per 10,000 pregnancies in Europe and a proportion of live-born patients between 75% and 83% [1,2]. CDH accounts for approximately 50 fatalities per year in Germany out of a total of 744,000 births per year (2015) [3]. Although prenatal diagnosis is feasible and would lead to intensified perinatal care, in Europe, approximately one out of four CDH cases is not detected prenatally [4].

There is no registry of CDH in Germany. Two German regional clinical registries for congenital malformations (Province Saxony-Anhalt, City of Mainz) contribute to the European network, EUROCAT [5]. These population-based registries focus on the epidemiology, prevalence, etiology, genetic diagnosis and risk factors for congenital malformations but not on the treatment modalities and associated outcomes.

Insurance companies increasingly evaluate their routine patient data, especially with respect to diagnoses, procedures, mortality and length of hospital stay. These datasets have been successfully used for epidemiological research [6,7,8].

There are detailed treatment guidelines for CDH patients [9,10,11]. Prenatally diagnosed neonates with CDH are already referred to specialized perinatal centres. However, there is still limited evidence that a higher caseload per centre results in a better outcome [12,13,14,15,16,17]. Selected NICU centres with ECMO (extracorporeal membrane oxygenation) facilities have published their outcome data on CDH patients [18,19,20,21,22] and established international networks [23,24,25]. In contrast, little is known about the overall outcome of patients with CDH in Germany.

Therefore, we analysed routine data from CDH patients insured by the largest local health insurance fund to evaluate treatment outcomes in the years 2009–2013.

## 2. Materials and Methods

### 2.1. Study Population

In Germany, in 2011, 87% of the inhabitants were insured under statutory health insurance (SHI) [26,27]. The Allgemeine Ortskrankenkasse (AOK) is the largest statutory health insurance company in the country; it has branches all over the country and covers approximately one third of German patients.

The number of live births from 2009 to 2013 was 665,126 (2009), 677,947 (2010), 662,685 (2011), 673,544 (2012) and 682,069 (2013) (total: 3,361,371) [3,28].

The number of newborns in the AOK insurance group in this period was estimated to be 1,043,029, resulting from a proportion of neonates insured by AOK of 31.0%.

### 2.2. Definitions

According to the International Classification of Diseases, tenth revision (ICD-10), datasets with the code for congenital diaphragmatic hernia (Q79.0) were reviewed. The ICD-10 code does not distinguish between the side or sizes of CDH. It is impossible to detect prenatal diagnosis of CDH using this classification.

Relevant additional ICD codes were screened in each individual case, and respective diagnoses were truncated and grouped together to facilitate analysis. The same was done for the German procedure classification codes (OPS). A list of relevant codes is provided in Table 1.

### 2.3. Data Extraction

The first screening for patients with CDH was performed by the research staff of the insurance company. All children born between 2009 and 2011 with the diagnosis Q79.0 who were admitted to the hospital at an age less than 28 days were selected. CDH, with a later presentation, could not be included. Any identifying information (i.e., name, the date of birth, the place of birth) was erased. No information about prenatal diagnosis, maternal health or the caseload of the perinatal treatment centre was provided. Concomitant diagnoses and codes for procedures (OPS), including the sex, year of birth and length of hospital stay, were documented for each patient. Anonymized data from CDH patients were sent to our institution for further analysis.

The data included information on survival at 4 time points (30 days, 3 months, 6 months and 12 months). We defined survival as being alive 12 months after discharge.

Of note, information on stillbirths and abortions due to CDH are not available from routine insurance data.

### 2.4. Statistical Considerations

We performed statistical analysis using R software for statistical computing, version 4.0.2 (R Foundation for Statistical Computing, Vienna, Austria, http://www.R-project.org/, accessed on 19 February 2021). We used the prodlim package to analyse the length of hospital stay. As the length of hospital stay was not censored, the Wilcoxon rank sum test was used for between-group comparisons. We applied the Wald test on logistic regression models including 95% confidence intervals to find possible risk factors for death from birth before the end of the first year after hospital discharge. All tests were two-sided, considering *p* < 0.05 to indicate statistical significance. The 95% confidence limits of the incidence were calculated according to the equation proposed by Bégau [29].

## 3. Results

A total of 288 cases were initially included, of which 3 had to be excluded due to a major inconsistency of the data (no mechanical ventilation, release from hospital within the first week without surgery). The remaining 285 patients were suitable for enrolment and analysis.

The final number of CDH patients (*n* = 285) in the screened population of 1,043,029 live births resulted in an incidence of 2.73 (2.42–3.06) per 10,000 live births (equal to 1:3700 live births). Overall mortality in this group was 30.2% (86/285), among this 87.2% (75/86) of fatalities occurring in the first 30 days of life and 72.1% (62/86) occurring before surgery.

A total of 78.2% (223/285) of patients underwent surgical repair. In this subgroup, the mortality rate was 10.8% (24/223). Only 8/22 patients who were not admitted on their first day of life to the treatment facility survived. This represented a mortality rate of 63.6% for this subgroup, which was significantly higher (*p* < 0.001).

**Figure 1:**
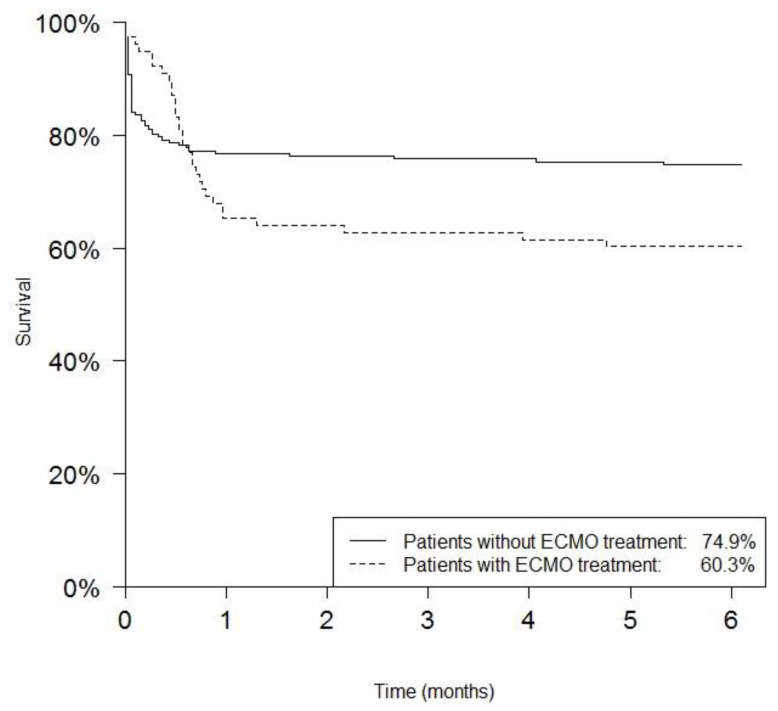
Survival of CDH patients in association with ECMO treatment and non-ECMO treatment in the first 6 months of life. The plot shows survival only until 6 months, since the vast majority of events occur in the first weeks, with a significant delay in the ECMO-subgroup.

A total of 67/285 patients (23.5%) were treated with ECMO. In this subpopulation, mortality was 41.8% (28/67, *p* = 0.019), with a median time to death of 18 days (range 2–143 days) compared to 2 days (range 1–212 days) in the group of non-ECMO patients. An overview of the mortality for the first 6 months of life for patients with CDH is given in Figure 1.

The median hospital stay was 7.5 days (range: 1–212 days, 1st quartile: 2 days, 3rd quartile: 19 days) for patients who died.

CDH patients frequently present with additional diagnoses, some of which are other malformations (i.e., heart defects) and others of which are a consequence of CDH (i.e., PPHN, lung hypoplasia). The number of additional diagnoses of the included cohort is given in Table 1.

**Figure 2:**
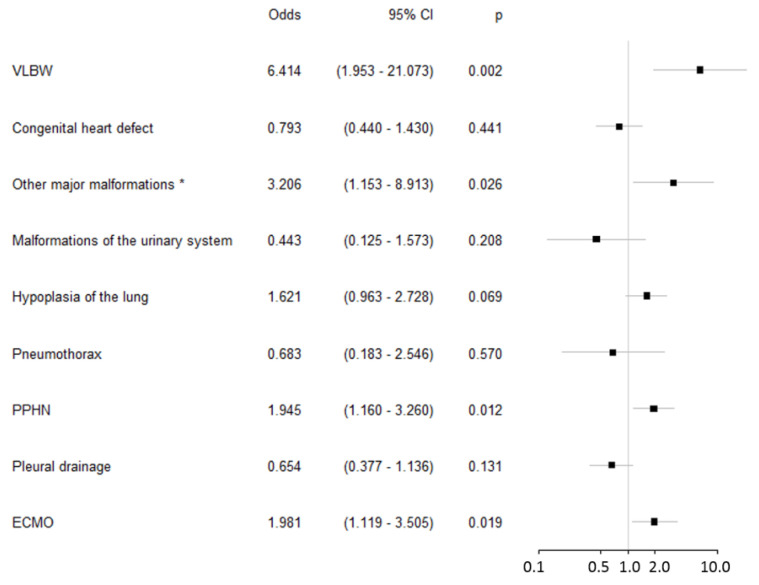
Risk factors for mortality in CDH patients (*n* = 285). * atresia of the small intestine (3 cases), atresia of the large intestine (3 cases), omphalocele (6 cases), and malformations of the spine (6 cases). PPHN: persistent pulmonary hypertension of the newborn, VLBW: very low birth weight, <1500 g.

Using these data, binominal logistic regression was used to identify risk factors for mortality within the first year of life (see Figure 2). These models identified very low birth weight (VLBW), extracorporeal membrane oxygenation (ECMO), persistent pulmonary hypertension (PPHN) and additional major malformations as relevant risk factors for mortality. There was one patient with chromosomal abnormality who died on the second day of life.

**Figure 3:**
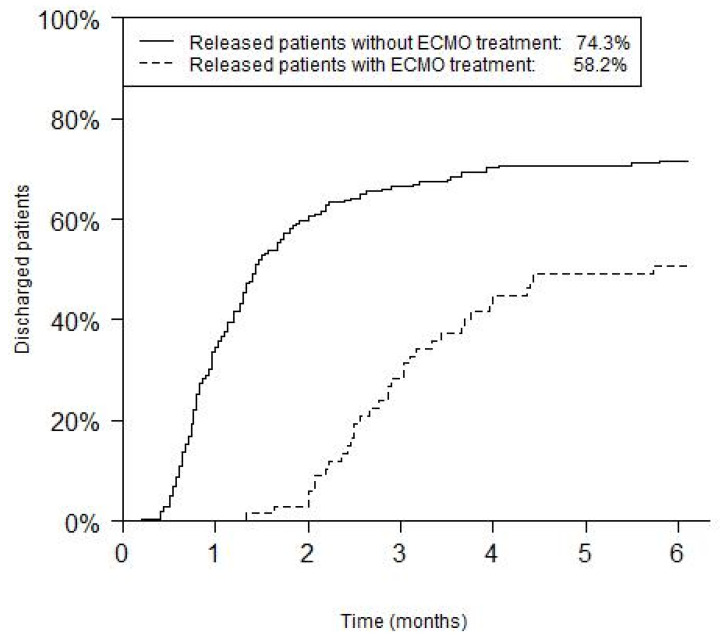
Total length of hospital stay, including re-admissions, of CDH-patients. Data are shown separately for the ECMO-treated and non-ECMO-treated patients (*p* value < 0.001).

Among the surviving patients, the median total length of hospital stay, including re-admissions (until 12 months after initial treatment) was 40 days (range: 6–592 days, 1st quartile: 24 days, 3rd quartile: 73.5 days). This was significantly different between the ECMO-treated (91 days, range: 40–592, 1st quartile: 73.5 days, 3rd quartile: 119.5 days) and non-ECMO-treated patients (32.5 days, range: 6–285 days, 1st quartile: 22 days, 3rd quartile: 50 days; *p* < 0.001, Figure 3).

## 4. Discussion

The birth prevalence of diaphragmatic hernia in the analysed population over five years was 2.73 per 10,000, which is in the upper range of previous European reports (2.3 (2.2 to 2.4) [1], 2.81 (2.65–2.97) [2] 2.79 (2.12–3.61) [30], per 10,000 births), since stillbirths and abortions are not included in our study. Looking at the reported incidence in our study, the use of routine data from the AOK seems to provide reliable numbers and substitutes for a national registry, which is clearly needed in some aspects. Currently, the setup of a national registry for several congenital anomalies including CDH has been finished and is under accreditation [31]. Established postnatal risk factors for worse outcome in CDH are low birth weight, low APGAR score, hypothermia, hypotension, pulmonary hypertension [32], low pO2, high pCO2 [33,34], and air leaks during treatment [35,36,37].

Our study confirmed very low birth weight, pulmonary hypertension and ECMO as factors associated with an unfavourable outcome (Figure 2). This has been clearly shown in the previous literature [32,34,35].

Two of the aforementioned risk factors, congenital heart disease and air leaks, were not significant predictors of mortality in our study (see Figure 2). We do not assume that congenital heart disease and air leaks are not relevant, but we would suspect that the specific coding may be insufficient. The provided information about heart disease summarizes the codes Q20–Q24 without discriminating between minor (i.e., ASD) and major defects (i.e., TGA). Furthermore, the provided codes for air leaks do not distinguish between a relevant preoperative pneumothorax and the common postoperative pneumothorax. Additionally, since routine data from health insurance do not include medical charts, we are not able to finally assess the influence of congenital heart disease and air leaks on the prognosis. This is a clear limitation of routine data acquisition.

The overall mortality of CDH patients in our observed population was 30.2%. In the literature, rates between 28% [32] and 62% [38] have been published for populations in developed countries [33,35,37,39,40,41]. In recent studies, lower mortality rates were reported in the UK and Ireland (16% until day 30 after surgery, 25% until one year of life) [42,43]. Mortality rate in a European multi-centre study of four high-volume centres (Mannheim, London, Rome and Rotterdam) was comparable (28.1%, until one year after discharge) [23] and the same was reported from large studies from the United States: 27% and 29% until discharge [44,45]; 28.8% and 32.5% until one year of life) [46,47].

Our CDH study reports a mortality rate of 9.8% in patients receiving surgical repair. Some further studies, which included also patients after surgical repair reported much lower mortality rates, ranging from 0% [21,48] and 5.1% [49] to 10.6% [50]. These low mortality rates were presumably found in these studies because open versus minimal-invasive surgical methods were compared in a pre-selected patient group. Furthermore, these data were provided from high-volume centres [21,48,49].

The largest ECMO centre for CDH patients in Germany has reported a mortality rate of 21.9% in their ECMO subgroup [23]. We found an overall higher mortality rate in our ECMO population. This effect might be related to poorer outcomes of these patients in other centres, may be due to other inclusion criteria for ECMO or different local treatment expertise. Groups with other associated malformations were also related to a higher mortality. However, due to the small numbers included, this might be a statistical effect only.

The use of ECMO in our cohort was 23.5%, which is higher than reported rates from unselected cohorts in other European countries: UK: 5% [42], France: 6% [41] but similar to reports from United States: 20% and 25% [17,35]. In the UK, there are five perinatal centres offering ECMO therapy for neonates and it is speculated, that the threshold to use this salvage therapy is here higher [51,52], may be in the British tradition to focus on evidence based and cost effective therapies [42].

Our analysis showed that patients who were admitted after their first day of life to a hospital with specialized care had a mortality that was as high as 64%. This subgroup might contain high-risk patients who were transferred postnatally to an ECMO centre or who were prenatally undetected CDH patients with a history of perinatal complications.

The median treatment duration until discharge according to recent publications is 31 days (excluding ECMO patients) [17], 32 days [45], 38 days [25], 39 days [41], and 39 days [53]. Re-hospitalizations are an additional burden [53,54] and reflect long-term morbidity [55]. Our study includes re-admissions after the first release from the hospital, which explains the longer treatment time in our study (40 days) and gives additional insights into the treatment course of CDH patients.

The difference in the median length of stay between ECMO-treated and non-ECMO-treated patients (Figure 3) seems to be trivial since only high-risk patients with severe lung hypoplasia are in need of extracorporeal membrane oxygenation.

### Limitations

The utilization of routine data from health insurances has the limitation, that over- or under-coding influences the data quality.

## 5. Conclusions

This is the largest cohort study of CDH patients from Germany covering an unselected patient group using data from statutory health insurance claims. Mortality of these CDH patients is comparable to that in previously published international data. The subgroup of patients who underwent ECMO showed a significantly higher mortality and longer hospital stay. However, a more specific analysis of risk factors would be dependent on more accurate and detailed coding, which underlines the need for a national CDH registry.

## Figures and Tables

**Table 1:** Concomitant diagnoses and procedures in 285 CDH patients (total), including 86 CDH fatalities.

**ICD Code(s)**	**Description**	**Number among 285 Patients (%)**	**Number among 86 Fatalities (%)**
J93	Pneumothorax	13 (4.6%)	3 (3.5%)
K56.2	Volvulus	1 (0.4%)	0
P07.00–P07.11	Very low birth weight (<1500 g)	14 (4.9%)	10 (11.6%)
Q20–Q24	Congenital heart defects	75 (26.3%)	20 (23.3%)
Q33.6	Congenital malformation of the lung, including hypoplasia	159 (55.8%)	55 (64%)
Q41	Congenital atresia or stenosis of the small intestine	3 (1.1%)	1 (1.2%)
Q42	Congenital atresia or stenosis of the large intestine	3 (1.1%)	3 (3.5%)
Q60–Q64	Congenital malformations of the urinary system	18 (6.3%)	3 (3.5%)
Q76	Congenital malformations of spine and bony thorax	6 (2.1%)	2 (2.3%)
Q79.2	Exomphalos	6 (2.1%)	3 (3.5%)
Q90–Q91	Trisomy 13, 18 and 21	2 (0.7%)	2 (2.3%)
**OPS-Code(s)**	**Description**	**Number among 285 patients (%)**	**Number among 86 fatalities (%)**
8.852	ECMO	67 (23.5%)	28 (32.6%)
5.340.0 & 8.144	Pleural drainage	98 (34.4%)	24 (27.9%)
5.431 & 5.464.21	Gastrostomy and/or jejunostomy	11 (3.9%)	7 (8.1%)

## References

[1] McGivern M.R., Best K.E., Rankin J., Wellesley D., Greenlees R., Addor M.-C., Arriola L., de Walle H., Barisic I., Beres J. (2015). Epidemiology of Congenital Diaphragmatic Hernia in Europe: A Register-Based Study. Arch. Dis. Child. Fetal Neonatal Ed..

[2] EUROCAT European Surveillance of Congenital Anomalies Cases and Prevalence (per 10,000 Births) of All Congenital Anomaly Subgroups for All Registries, from 2008–2012. https://eu-rd-platform.jrc.ec.europa.eu.

[3] (2017). Statistisches Jahrbuch Deutschland 2017. Statistical Yearbook Germany 2017.

[4] EUROCAT Prenatal Detection Rates 2011–2015. http://www.eurocat-network.eu/newprevdata/showPDF.aspx?winx=1256&winy=884&file=pd1.aspx.

[5] Calzolari E., Barisic I., Loane M., Morris J., Wellesley D., Dolk H., Addor M.-C., Arriola L., Bianchi F., Neville A.J. (2014). Epidemiology of Multiple Congenital Anomalies in Europe: A EUROCAT Population-Based Registry Study. Birt. Defects Res. A Clin. Mol. Teratol..

[6] Gilfrich C., Leicht H., Fahlenbrach C., Jeschke E., Popken G., Stolzenburg J.U., Weißbach L., Zastrow C., Günster C. (2016). Morbidity and Mortality after Surgery for Lower Urinary Tract Symptoms: A Study of 95 577 Cases from a Nationwide German Health Insurance Database. Prostate Cancer Prostatic Dis..

[7] Jeschke E., Searle J., Günster C., Baberg H.T., Dirschedl P., Levenson B., Malzahn J., Mansky T., Möckel M. (2017). Drug-Eluting Stents in Clinical Routine: A 1-Year Follow-up Analysis Based on German Health Insurance Administrative Data from 2008 to 2014. BMJ Open.

[8] Schmedding A., Wittekind B., Salzmann-Manrique E., Schloesser R., Rolle U. (2020). Decentralized Surgery of Abdominal Wall Defects in Germany. Pediatr. Surg. Int..

[9] Kilian A.K., Schaible T., Hofmann V., Brade J., Neff K.W., Büsing K.A. (2009). Congenital Diaphragmatic Hernia: Predictive Value of MRI Relative Lung-to-Head Ratio Compared with MRI Fetal Lung Volume and Sonographic Lung-to-Head Ratio. AJR Am. J. Roentgenol..

[10] Reiss I., Schaible T., van den Hout L., Capolupo I., Allegaert K., van Heijst A., Gorett Silva M., Greenough A., Tibboel D., CDH EURO Consortium (2010). Standardized Postnatal Management of Infants with Congenital Diaphragmatic Hernia in Europe: The CDH EURO Consortium Consensus. Neonatology.

[11] Snoek K.G., Reiss I.K.M., Greenough A., Capolupo I., Urlesberger B., Wessel L., Storme L., Deprest J., Schaible T., van Heijst A. (2016). Standardized Postnatal Management of Infants with Congenital Diaphragmatic Hernia in Europe: The CDH EURO Consortium Consensus—2015 Update. Neonatology.

[12] Grushka J.R., Laberge J.-M., Puligandla P., Skarsgard E.D. (2009). Canadian Pediatric Surgery Network Effect of Hospital Case Volume on Outcome in Congenital Diaphragmatic Hernia: The Experience of the Canadian Pediatric Surgery Network. J. Pediatr. Surg..

[13] Freeman C.L., Bennett T.D., Casper T.C., Larsen G.Y., Hubbard A., Wilkes J., Bratton S.L. (2014). Pediatric and Neonatal Extracorporeal Membrane Oxygenation: Does Center Volume Impact Mortality?. Crit. Care Med..

[14] Harting M.T., Hollinger L., Tsao K., Putnam L.R., Wilson J.M., Hirschl R.B., Skarsgard E.D., Tibboel D., Brindle M.E., Lally P.A. (2018). Aggressive Surgical Management of Congenital Diaphragmatic Hernia: Worth the Effort?: A Multicenter, Prospective, Cohort Study. Ann. Surg..

[15] Bucher B.T., Guth R.M., Saito J.M., Najaf T., Warner B.W. (2010). Impact of Hospital Volume on In-Hospital Mortality of Infants Undergoing Repair of Congenital Diaphragmatic Hernia. Ann. Surg..

[16] Apfeld J.C., Kastenberg Z.J., Gibbons A.T., Carmichael S.L., Lee H.C., Sylvester K.G. (2020). Treating Center Volume and Congenital Diaphragmatic Hernia Outcomes in California. J. Pediatr..

[17] Sømme S., Shahi N., McLeod L., Torok M., McManus B., Ziegler M.M. (2019). Neonatal Surgery in Low- vs. High-Volume Institutions: A KID Inpatient Database Outcomes and Cost Study after Repair of Congenital Diaphragmatic Hernia, Esophageal Atresia, and Gastroschisis. Pediatr. Surg. Int..

[18] Beck C., Alkasi O., Nikischin W., Engler S., Caliebe A., Leuschner I., von Kaisenberg C.S. (2008). Congenital Diaphragmatic Hernia, Etiology and Management, a 10-Year Analysis of a Single Center. Arch. Gynecol. Obstet..

[19] Hofmann S.R., Stadler K., Heilmann A., Häusler H.J., Fitze G., Kamin G., Nitzsche K.I., Hahn G., Dinger J. (2012). Stabilisation of Cardiopulmonary Function in Newborns with Congenital Diaphragmatic Hernia Using Lung Function Parameters and Hemodynamic Management. Klin. Padiatr..

[20] Proquitté H., Freiberger O., Yilmaz S., Bamberg C., Degenhardt P., Roehr C.C., Wauer R.R., Schmalisch G. (2010). The Effect of Surgery on Lung Volume and Conventional Monitoring Parameters in Ventilated Newborn Infants. Eur. Respir. J..

[21] Szavay P.O., Obermayr F., Maas C., Luenig H., Blumenstock G., Fuchs J. (2012). Perioperative Outcome of Patients with Congenital Diaphragmatic Hernia Undergoing Open versus Minimally Invasive Surgery. J. Laparoendosc. Adv. Surg. Tech. A.

[22] Kipfmueller F., Schroeder L., Berg C., Heindel K., Bartmann P., Mueller A. (2018). Continuous Intravenous Sildenafil as an Early Treatment in Neonates with Congenital Diaphragmatic Hernia. Pediatr. Pulmonol..

[23] Snoek K.G., Greenough A., van Rosmalen J., Capolupo I., Schaible T., Ali K., Wijnen R.M., Tibboel D. (2018). Congenital Diaphragmatic Hernia: 10-Year Evaluation of Survival, Extracorporeal Membrane Oxygenation, and Foetoscopic Endotracheal Occlusion in Four High-Volume Centres. Neonatology.

[24] Kipfmueller F., Heindel K., Schroeder L., Berg C., Dewald O., Reutter H., Bartmann P., Mueller A. (2018). Early Postnatal Echocardiographic Assessment of Pulmonary Blood Flow in Newborns with Congenital Diaphragmatic Hernia. J. Perinat. Med..

[25] Putnam L.R., Harting M.T., Tsao K., Morini F., Yoder B.A., Luco M., Lally P.A., Lally K.P. (2016). Congenital Diaphragmatic Hernia Study Group Congenital Diaphragmatic Hernia Defect Size and Infant Morbidity at Discharge. Pediatrics.

[26] Federal Statistical Office GENESIS-Online: 12411-0001: [Bevölkerung: Deutschland, Stichtag] Population: Germany, Deadline. https://www-genesis.destatis.de/genesis//online?operation=table&code=12411-0001&bypass=true&levelindex=0&levelid=1610283403187#abreadcrumb.

[27] Federal Ministry of Health [Mitglieder und Versicherte der Gesetzlichen Krankenversicherung (GKV)] Members and Insured Persons in Statutory Health. https://www.bundesgesundheitsministerium.de/themen/krankenversicherung/zahlen-und-fakten-zur-krankenversicherung/mitglieder-und-versicherte.html.

[28] (2011). Statistisches Jahrbuch Für Die Bundesrepublik Deutschland. Statistical Yearbook for the Federal Republic of Germany.

[29] Bégaud B., Martin K., Abouelfath A., Tubert-Bitter P., Moore N., Moride Y. (2005). An Easy to Use Method to Approximate Poisson Confidence Limits. Eur. J. Epidemiol..

[30] Götz D. Annual Report 2014 of the Federal State of Saxony-Anhalt about the Frequency of Congenital Malformations and Anomalies as Well as Chromosomal Aberrations. http://www.angeborene-fehlbildungen.com/monz_mm/Bericht_2014_engl.pdf.

[31] Schmedding A., Rolle U. (2020). Qualitätsmessung bei seltenen Erkrankungen—KinderRegister für angeborene Fehlbildungen—BDC|Online. Passion Chir..

[32] Brindle M.E., Cook E.F., Tibboel D., Lally P.A., Lally K.P. (2014). Congenital Diaphragmatic Hernia Study Group A Clinical Prediction Rule for the Severity of Congenital Diaphragmatic Hernias in Newborns. Pediatrics.

[33] Jaillard S.M., Pierrat V., Dubois A., Truffert P., Lequien P., Wurtz A.J., Storme L. (2003). Outcome at 2 Years of Infants with Congenital Diaphragmatic Hernia: A Population-Based Study. Ann. Thorac. Surg..

[34] Skarsgard E.D., MacNab Y.C., Qiu Z., Little R., Lee S.K. (2005). Canadian Neonatal Network SNAP-II Predicts Mortality among Infants with Congenital Diaphragmatic Hernia. J. Perinatol..

[35] Aly H., Bianco-Batlles D., Mohamed M.A., Hammad T.A. (2010). Mortality in Infants with Congenital Diaphragmatic Hernia: A Study of the United States National Database. J. Perinatol..

[36] Boloker J., Bateman D.A., Wung J.-T., Stolar C.J.H. (2002). Congenital Diaphragmatic Hernia in 120 Infants Treated Consecutively with Permissive Hypercapnea/Spontaneous Respiration/Elective Repair. J. Pediatr. Surg..

[37] Colvin J., Bower C., Dickinson J.E., Sokol J. (2005). Outcomes of Congenital Diaphragmatic Hernia: A Population-Based Study in Western Australia. Pediatrics.

[38] Tennant P.W.G., Pearce M.S., Bythell M., Rankin J. (2010). 20-Year Survival of Children Born with Congenital Anomalies: A Population-Based Study. Lancet Lond. Engl..

[39] Burgos C.M., Frenckner B., Luco M., Harting M.T., Lally P.A., Lally K.P., Congenital Diaphragmatic Hernia Study Group (2017). Right versus Left Congenital Diaphragmatic Hernia—What’s the Difference?. J. Pediatr. Surg..

[40] Gallot D., Boda C., Ughetto S., Perthus I., Robert-Gnansia E., Francannet C., Laurichesse-Delmas H., Jani J., Coste K., Deprest J. (2007). Prenatal Detection and Outcome of Congenital Diaphragmatic Hernia: A French Registry-Based Study. Ultrasound Obstet. Gynecol..

[41] Barrière F., Michel F., Loundou A.D., Fouquet V., Kermorvant E., Blanc S., Carricaburu E., Desrumaux A., Pidoux O., Arnaud A. (2018). One-Year Outcome for Congenital Diaphragmatic Hernia: Results From the French National Register. J. Pediatr..

[42] Long A.-M., Bunch K.J., Knight M., Kurinczuk J.J., Losty P.D. (2018). BAPS-CASS Early Population-Based Outcomes of Infants Born with Congenital Diaphragmatic Hernia. Arch. Dis. Child. Fetal Neonatal Ed..

[43] Long A.-M., Bunch K.J., Knight M., Kurinczuk J.J., Losty P.D. (2019). BAPS-CASS One-Year Outcomes of Infants Born with Congenital Diaphragmatic Hernia: A National Population Cohort Study. Arch. Dis. Child. Fetal Neonatal Ed..

[44] Murthy K., Pallotto E.K., Gien J., Brozanski B.S., Porta N.F.M., Zaniletti I., Keene S., Chicoine L.G., Rintoul N.E., Dykes F.D. (2016). Predicting Death or Extended Length of Stay in Infants with Congenital Diaphragmatic Hernia. J. Perinatol..

[45] Grover T.R., Murthy K., Brozanski B., Gien J., Rintoul N., Keene S., Najaf T., Chicoine L., Porta N., Zaniletti I. (2015). Short-Term Outcomes and Medical and Surgical Interventions in Infants with Congenital Diaphragmatic Hernia. Am. J. Perinatol..

[46] Ramakrishnan R., Salemi J.L., Stuart A.L., Chen H., O’Rourke K., Obican S., Kirby R.S. (2018). Trends, Correlates, and Survival of Infants with Congenital Diaphragmatic Hernia and Its Subtypes. Birth Defects Res..

[47] Shanmugam H., Brunelli L., Botto L.D., Krikov S., Feldkamp M.L. (2017). Epidemiology and Prognosis of Congenital Diaphragmatic Hernia: A Population-Based Cohort Study in Utah. Birth Defects Res..

[48] Costerus S., Zahn K., van de Ven K., Vlot J., Wessel L., Wijnen R. (2016). Thoracoscopic versus Open Repair of CDH in Cardiovascular Stable Neonates. Surg. Endosc..

[49] Putnam L.R., Tsao K., Lally K.P., Blakely M.L., Jancelewicz T., Lally P.A., Harting M.T., Congenital Diaphragmatic Hernia Study Group and the Pediatric Surgery Research Collaborative (2017). Minimally Invasive vs. Open Congenital Diaphragmatic Hernia Repair: Is There a Superior Approach?. J. Am. Coll. Surg..

[50] Duess J.W., Zani-Ruttenstock E.M., Garriboli M., Puri P., Pierro A., Hoellwarth M.E. (2015). Outcome of Right-Sided Diaphragmatic Hernia Repair: A Multicentre Study. Pediatr. Surg. Int..

[51] Wang Y., Honeyford K., Aylin P., Bottle A., Giuliani S. (2019). One-Year Outcomes for Congenital Diaphragmatic Hernia. BJS Open.

[52] Lally K.P. (2016). Congenital Diaphragmatic Hernia—The Past 25 (or so) Years. J. Pediatr. Surg..

[53] Wynn J., Aspelund G., Zygmunt A., Stolar C.J.H., Mychaliska G., Butcher J., Lim F.-Y., Gratton T., Potoka D., Brennan K. (2013). Developmental Outcomes of Children with Congenital Diaphragmatic Hernia: A Multicenter Prospective Study. J. Pediatr. Surg..

[54] Benoist G., Mokhtari M., Deschildre A., Khen-Dunlop N., Storme L., Benachi A., Delacourt C. (2016). Risk of Readmission for Wheezing during Infancy in Children with Congenital Diaphragmatic Hernia. PLoS ONE.

[55] Bagolan P., Morini F. (2007). Long-Term Follow up of Infants with Congenital Diaphragmatic Hernia. Semin. Pediatr. Surg..

